# Classification of Sleep Apnea Based on Sub-Band Decomposition of EEG Signals

**DOI:** 10.3390/diagnostics11091571

**Published:** 2021-08-30

**Authors:** Rajeswari Jayaraj, Jagannath Mohan

**Affiliations:** School of Electronics Engineering, Vellore Institute of Technology (VIT) Chennai, Vellore 600127, Tamil Nadu, India; rajeswari.j2015@vit.ac.in

**Keywords:** EEG signals, wavelet packet decomposition, brain perfusion, neural activity, support vector machine

## Abstract

To classify between normal and sleep apnea subjects based on sub-band decomposition of electroencephalogram (EEG) signals. This study comprised 159 subjects obtained from the ISRUC (Institute of System and Robotics—University of Coimbra), Sleep-EDF (European Data Format), and CAP (Cyclic Alternating Pattern) Sleep database, which consists of normal and sleep apnea subjects. The wavelet packet decomposition method was incorporated to categorize the EEG signals into five frequency bands, namely, alpha, beta, delta, gamma, and theta. Entropy and energy (non-linear) for all bands was calculated and as a result, 10 features were obtained for each EEG signal. The ratio of EEG bands included four parameters, including heart rate, brain perfusion, neural activity, and synchronization. In this study, a support vector machine with kernels and random forest classifiers was used for classification. The performance measures demonstrated that the improved results were obtained from the support vector machine classifier with a kernel polynomial order 2. The accuracy (90%), sensitivity (100%), and specificity (83%) with 14 features were estimated using the data obtained from ISRUC database. The proposed study is feasible and seems to be accurate in classifying the subjects with sleep apnea based on the extracted features from EEG signals using a support vector machine classifier.

## 1. Introduction

Sleep disorder is categorized into sleep apnea, narcolepsy, insomnia, and nightmare syndrome. Sleep apnea is a serious disorder and one of the major causes of cardiovascular disease, stroke, and heart disease. ET health world reported that in India, 93% of men and 82% of women have untreated sleep apnea [1]. Early diagnoses and treatment can improve the health conditions of subjects with sleep apnea. According to the guidance of American Academy of Sleep Medicine (AASM), the conventional polysomnography (PSG) method is used for diagnosing sleep apnea from electrocardiography (ECG), electroencephalography (EEG), electromyography (EMG), electrooculography (EOG), respiratory effort, nasal airflow, and oxygen levels [2]. The 30 s epochs are utilized to score the PSG recordings. Apnea is the complete cessation of oronasal flow (≥10 s), whereas hypopnea is a reduction in the respiratory airflow (≥30%). The severity of obstructive sleep apnea (OSA) is measured using the apnea–hypopnea index (AHI) or respiratory disturbance index (RDI). The AHI index is defined as the sum of apnea and hypopnea episodes divided by the total sleep time. This index varies into normal (<5/h), mild OSA (5–14.9/h), moderate OSA (15–29.9/h), and severe OSA (≥30/h) [3]. The difference between PSG and the respiratory polygraphy (RP) method was investigated by Tan et al. [4]. Lab respiratory polygraphy uses the same equipment and procedure as PSG does except for the recording montage (EEG, EMG, and EOG signals). The thermistor and nasal pressure transducer signals are deviated in home-based respiratory polygraphy. In conclusion, the performance of PSG is high compared with home-based respiratory polygraphy. As the PSG method is complex and time-consuming, numerous studies focused on an ECG-based diagnosing system [5,6]. EEG signal processing is required to analyze the activity of the brain and diagnose the normal and abnormal activities of any disease. Few research works carried out sleep stage classification using EEG signals. Cui et al. [7] propounded the convolutional neural network (CNN) and fine-grained segments for automatic sleep stage classification. This method consists of max-pooling layers and two convolutional layers with unique designed features.

Another study used degree distribution, a horizontal visual graph, and difference visual graph features and portrayed an improvement with an accuracy of 87.5% in six stages of sleep classification from EEG signals [8]. A three-band time-frequency localized (TBTFL) wavelet filter bank has been utilized for sleep stage classification from EEG signals using the features such as sample entropy, signal fractal dimensions, and log energy. Support vector machine (SVM), K-nearest neighbor (KNN), and complex tress classifiers were used for classification. The three-level wavelet decomposition method segregated the EEG signals into seven sub-bands. [9]. Tzimourta et al. [10] proposed a five-stage classification method using EEG signals and obtained promising results from a random forest (RF) classifier compared with other classifiers such as KNN, SVM, decision tree, and Naive Bayes (NB). Another study suggested a sleep stage classification from the EEG signal using stock well transform. This study utilized SVM, KNN, and ensemble bagged tree classifiers [11]. Savareh et al. [12] performed a sleep stage classification from wavelet tree features using SVM and an artificial neural network (ANN). A recent literature review demonstrated more suitable preprocessing techniques, feature selection, and classification methods for sleep stage classification [13]. The sleep stages were explored by Hertenstein et al. [14] in healthy subjects. The investigation was performed using the spectral analysis of EEG signals based on age and gender. Furthermore, the sleep quality was examined using EEG signals from healthy subjects [15]. The results demonstrated that the beta range is high in adults. The limitation of the current study is the subject selection without any validation, and assumes the sleep apnea and normal subjects as the categories already mentioned in the existing database.

Elwali et al. [16] designed a screening method for OSA using anthropometric features and tracheal breathing sounds with the support of an RF classifier. Few existing studies have significantly applied the EEG signals and machine learning approaches for sleep apnea detection. In addition, the EEG signal band segregation was accomplished for better performance. Consequently, EEG signals were classified into alpha, beta, delta, gamma, and theta. The frequency range of each band was different, and was given as follows: the delta lay below 4 Hz, theta lay between 4 Hz and 8 Hz, alpha lay between 8 Hz and 13 Hz, beta lay between 14 Hz and 32 Hz, and gamma lay above 32 Hz. The WPD method was used for EEG signal band segregation and achieved results with better accuracy [17]. Almuhammadi et al. [18] demonstrated a classification approach using SVM, ANN, NB, and linear discriminant analysis (LDA) methods. The infinite impulse Butterworth bandpass filter was used for preprocessing. Energy and variance were calculated for each band to classify normal and sleep subjects. Zhao et al. [19] used SVM, KNN, and RF for OSA, central sleep apnea (CSA), and normal breathing classification. Sample entropy and variance were manipulated and fed into classifiers. Furthermore, the inter-band energy ratio was used to diagnose sleep apnea using SVM, KNN, LDA, and NB classifiers [20]. The energy ratios were delta-theta (δ-θ), delta-alpha (δ-α), delta-theta (δ-σ), delta-beta (δ-β), and theta-alpha (θ-α). Nagendra et al. [21] investigated the effects of yoga practice in young normal subjects using the parameters of ECG and EEG bands. The measurement was performed using the EEG frequency band ratios and better outcomes were achieved using various cognitive functions.

Most of the existing research studies have demonstrated sleep stage classification from EEG signals using machine learning approaches. Multiple types of literature works have utilized ECG signals for sleep apnea detection and demonstrated the potential results. Only a few studies discussed the detection of sleep apnea based on EEG signals, which motivated us to use EEG signals for sleep apnea detection with SVM and RF classifier for classification. The proposed method consists of the notch filter for preprocessing and WPD for EEG signal band segregation. In addition, this study includes the non-linear and ratios of frequency band parameters for feature extraction. Here, the features are calculated using EEG bands and applied to the input for both SVM and RF classifiers. Finally, the proposed work demonstrated the classification comparison between three publicly available databases and documented the significant performance analysis.

## 2. Materials and Methods

### 2.1. Database Description

#### 2.1.1. ISRUC Sleep Database

This study comprised a total of 89 subjects from the publicly available database of the Institute of System and Robotics—University of Coimbra (ISRUC). There was an assortment of three subgroups. Group 1 and group 2 consisted of both sleep apnea and normal subjects. Group 3 had the normal subjects. In this study, EEG signals were acquired from the C3-A2 electrode location [22]. A total of 57 sleep apnea subjects and 32 normal subjects from group 1 and group 3 were utilized in this study, which involved 45 male and 44 female subjects. Adults between 20 and 85 years (mean—51, standard deviation—16) were in three groups.

#### 2.1.2. Sleep—EDF Database

A total of 40 subjects were used in this study, 20 from sleep cassette and 20 from sleep telemetry, including 31 female and 9 male subjects aged between 26 and 51 years (mean—36.8 years, standard deviation—14.68). The EEG signals were recorded using the locations of both Fpz-Cz and Pz-Oz electrodes at 30 s epochs [23].

#### 2.1.3. CAP Sleep Database

A total of 20 subjects were used in this study, which involved 16 normal subjects and 4 sleep disorder subjects. Nine females and 11 males aged between 25 and 78 years (mean—51.5, standard deviation—37.47) subjects classified as both normal and sleep disordered were utilized in this study [24]. The EEG signals were recorded from C3-A2 and C4-A1 locations.

**Figure 1:**
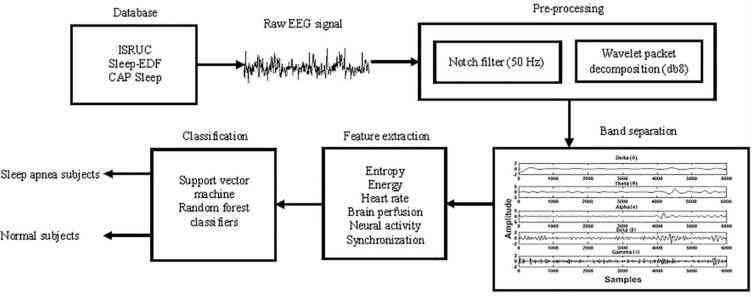
Proposed architecture diagram to classify normal and sleep apnea subjects.

Figure 1 shows the proposed architecture diagram to classify normal and sleep apnea subjects. The demographic characteristics of the ISRUC, Sleep-EDF, and Cap Sleep databases are represented in Table 1.

### 2.2. Pre-Processing and Band Separation

This study used a 50 Hz notch filter to suppress the unwanted AC voltage line from 30 s epoch raw EEG signals. Furthermore, the WPD method was used to decompose the EEG signals into approximation and detail coefficients at a higher level. The Daubechies mother wavelet (dbN) was used for EEG frequency band partitioning [25]. WPD generates 2n coefficients for n levels of decomposition. WPD was calculated using Equation (1).

(1) $$C_{p}^{n,j}=\int_{-\infty }^{\infty }x\left(t\right)\psi_{n}\left(2^{-j}-p\right)\mathrm{dt}$$

where x(t) is the EEG signal, C is the coefficient of WPD, p is the position parameter, ψ_n_ is the wavelets, n is the channel number, and j is the number of decomposition levels. In addition, the approximation and detail coefficients were calculated for all EEG bands: alpha (α), beta (β), theta (θ), gamma (γ), and delta (δ). Here, three-level WPD was performed to develop a binary tree (23 = 8) [26]. The proposed study used the detail coefficients to calculate the feature values.

### 2.3. Feature Extraction Method of the Proposed Study

This study attempted to use features such as entropy, energy, heart rate (HR), brain perfusion, neural activity, and synchronization, which manipulate the ratios of the frequency band and develop a suitable set of values for classifiers. Entropy and energy are the most familiar features utilized for sleep apnea classification [27,28,29]. The entropy and energy features were calculated for five EEG bands in both approximation and detail coefficients. Entropy (H) was computed by Equation (2). The energy (E) was calculated using Equation (3).

(2) $$H=-\overset{N-1}{\underset{i=0}{\sum }}{\left(p_{i}\right)}^{2}\log{\left(p_{i}\right)}^{2}$$

(3) $$E=\overset{N-1}{\underset{i=0}{\sum }}p_{i}^{2}$$

where pi is the wavelet detailed coefficients at level i and N is the total number of decomposition levels. The EEG frequency band ratios were interpreted, including heart rate, brain perfusion, neural activity, and synchronization, in Equations (4)–(7) [20,21]. The heart rate is the ratio corresponding to the physiological condition of the relaxed state, whereas the brain perfusion indicates the relaxed state, which increased in parietal and temporal and decreased in frontal and occipital lobes. Similarly, the neural activity elaborates the representation of improvement in cognitive skills and the synchronization is for analyzing the correlation of deactivated cortical [30].

(4) Heart rate=θ/α

(5) Brain perfusion=α/δ

(6) Neural activity=β/θ

(7) Synchronization=δ/θ

Ten features were computed using entropy and energy for each EEG band, and four features from the EEG band ratios. In comparison with recent literature, this study obtained a maximum of 14 features for each EEG signal and provided results with better accuracy. The extracted feature values of sleep apnea and normal subjects for five EEG bands were demonstrated using a box plot obtained from the ISRUC, Sleep-EDF, and CAP Sleep databases.

### 2.4. Classification Module

#### 2.4.1. Support Vector Machine Classifier with Kernels

In this study, the normal and sleep apnea subjects were the two classes of input. The SVM is a binary classifier used to separate the two classes using the hyperplane with the highest margin. It utilizes a large set of points to develop a decision boundary, which are support vectors [31]. Different kernel types are used, such as sigmoid, linear, polynomial, and radial basis function (RBF). The proposed study consisted of the linear, RBF, and polynomial order 2 and 3 kernels for classification and obtained improved accuracy in polynomial order 2. The experimental setup consisted of training and testing algorithms, and each kernel had separate training and testing data. The report demonstrates that the increased training data (90%) and decreased testing data (10%) provided results with better accuracy [7]. This study followed a similar percentage of data for performance analysis.

#### 2.4.2. Random Forest Classifier

Another method utilized in this proposed work was the RF classifier. It is a fast feature selection method among multiple feature sets. The measurement of this classifier is made when the out-of-bag (OOB) values of features are permuted using the misclassification rate [32]. The OOB error is measured when each classification tree has been built. It measures the error of overall trained classification trees. As mentioned above for SVM, the features are segregated into training and testing data. It is a supervised learning algorithm that develops a decision tree on the randomly preferred database. The results of the multiple decision trees are combined by bootstrap-aggregated decision trees to reduce overfitting, which improves the generalization error. A prediction is made for each tree and the best one is selected by voting [33]. This classifier seems to be an efficient method because it is suitable for large databases. To validate the performance of the classifier, this study used a 10-fold cross-validation method, which is a robust model that has a minimum amount of redundancy and overfitting.

### 2.5. Performance Analysis

To evaluate the performance metrics, the accuracy, specificity, and sensitivity were calculated in this study (Equations (8)–(10)) [17].

(8) $$\mathrm{Accuracy}=\frac{\mathrm{TP}+\mathrm{TN}}{\mathrm{TP}+\mathrm{TN}+\mathrm{FP}+\mathrm{FN}}$$

(9) $$\mathrm{Specificity}=\frac{\mathrm{TN}}{\mathrm{TN}+\mathrm{FP}}$$

(10) $$\mathrm{Sensitivity}=\frac{\mathrm{TP}}{\mathrm{TP}+\mathrm{FN}}$$

where True Positive (TP) is the correct number of subjects recognized as sleep apnea, False Positive (FP) is the normal subjects wrongly recognized as sleep apnea, True Negative (TN) is the number of subjects recognized as normal, and False Negative (FN) is the number of sleep apnea subjects wrongly recognized as normal.

## 3. Results

**Figure 2:**
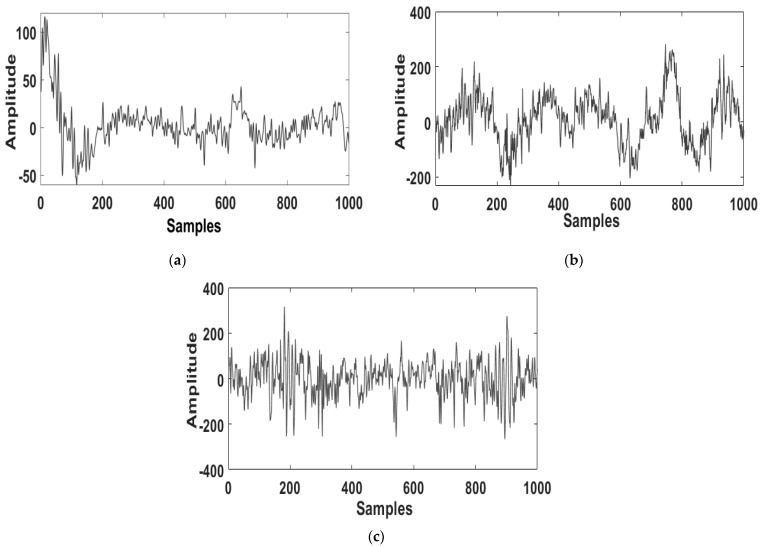
Typical raw EEG signals of subjects with sleep apnea obtained from the (**a**) ISRUC, (**b**) Sleep-EDF, and (**c**) CAP Sleep databases.

**Figure 3:**
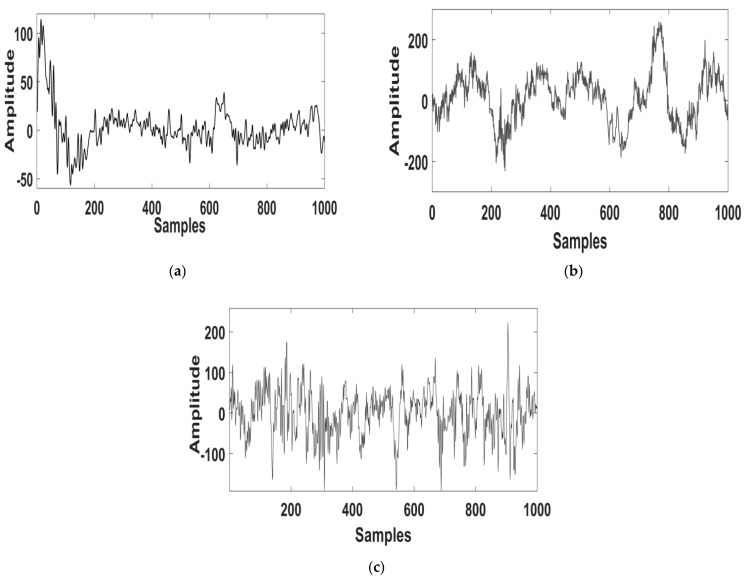
Typical filtered EEG signals of subjects with sleep apnea obtained from the (**a**) ISRUC, (**b**) Sleep-EDF, and (**c**) CAP Sleep databases.

The analysis deployed a MATLAB tool for processing the EEG signals. Figure 2a–c shows the raw EEG signal with a sampling frequency of 200 Hz obtained from the ISRUC, 100 Hz from the Sleep-EDF, and 512 Hz from the CAP Sleep database. The signal was then filtered by a notch filter that performed efficient noise removal. The outcome of the filtered EEG signal is depicted in Figure 3a–c.

**Figure 4:**
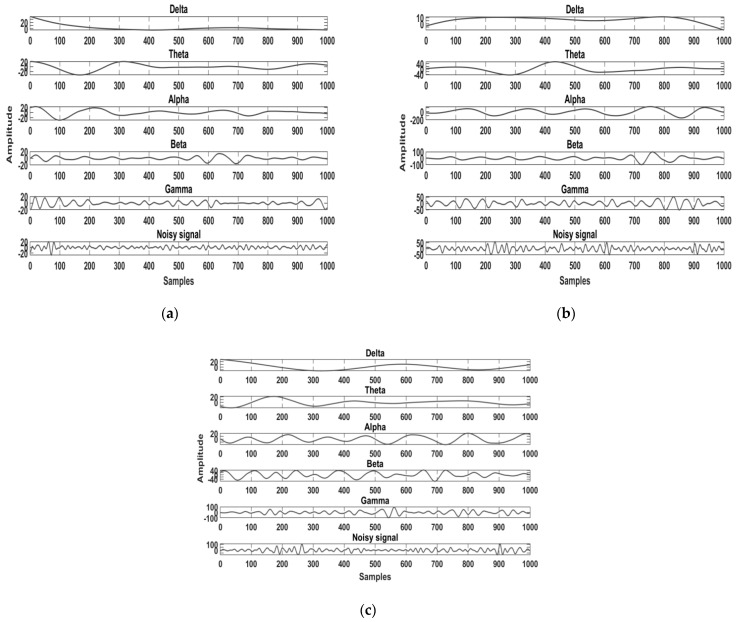
Band separation of sleep apnea subjects obtained from the (**a**) ISRUC database, (**b**) Sleep-EDF database, and (**c**) CAP Sleep database.

After the preprocessing, the Daubechies wavelet (db8) was used to partition the filtered EEG signals into approximation and detail coefficients for five bands, including alpha, beta, delta, gamma, and theta. Each database was processed by a different sampling frequency, which is given as follows: for the ISRUC database the signal processed at a frequency of 200 Hz [22], the Sleep-EDF database at 100 Hz [23], and the CAP Sleep database at 512 Hz [24]. The compressed representation of EEG signal is demonstrated by the extracted wavelet coefficients shown in Figure 4a–c. The approximation coefficients and detail coefficients for the five bands were obtained and each band of detail coefficients was considered for feature extraction.

**Figure 5:**
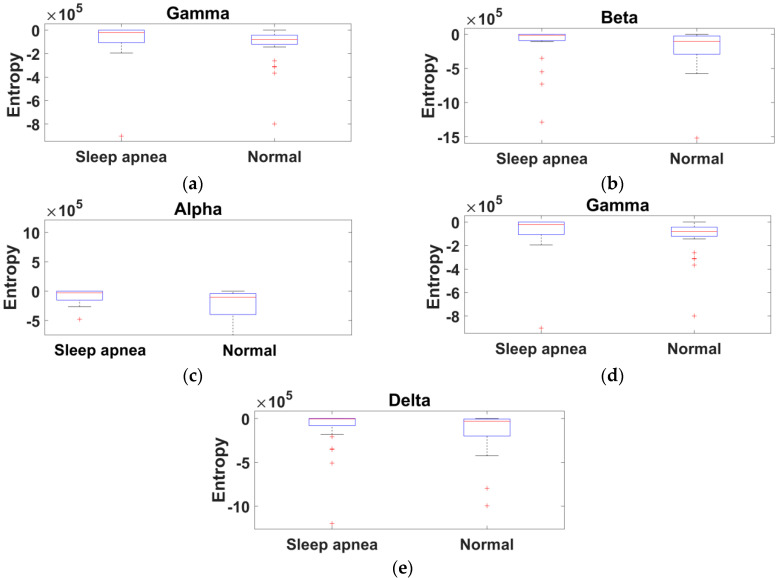
Box-whisker plot representation of entropy obtained from the ISRUC database: (**a**) gamma band, (**b**) beta band, (**c**) alpha band, (**d**) theta band, and (**e**) delta band.

**Figure 6:**
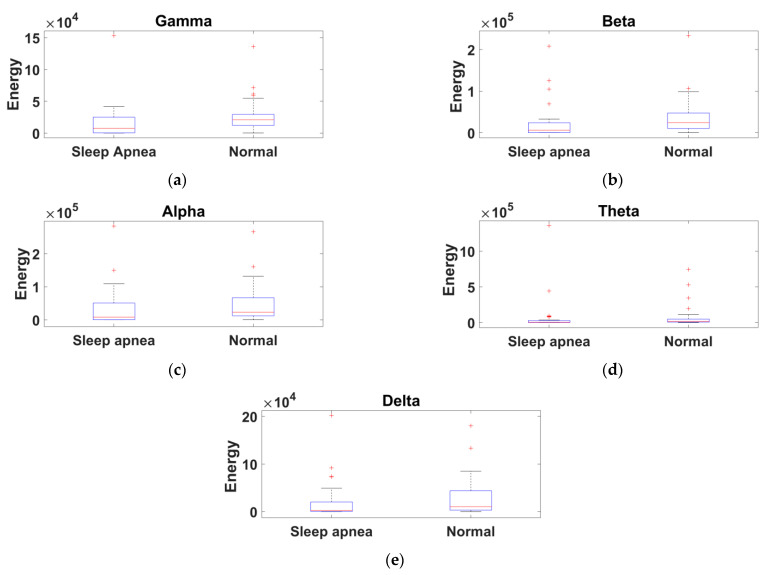
Box-whisker plot representation of the energy obtained from the ISRUC database: (**a**) gamma band, (**b**) beta band, (**c**) alpha band, (**d**) theta band, and (**e**) delta band.

**Figure 7:**
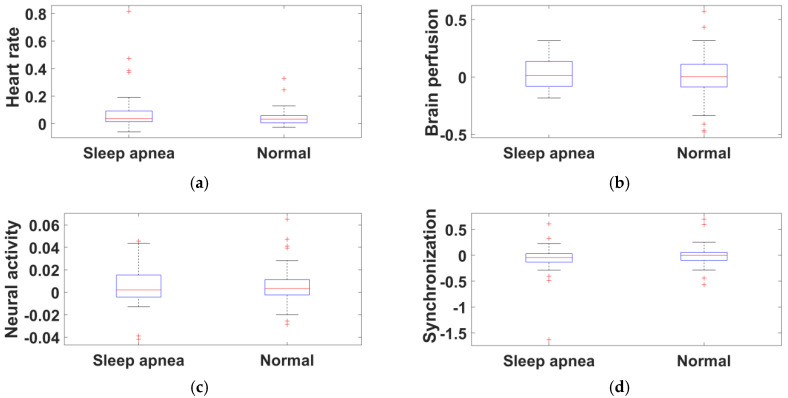
Box-whisker plot representation of frequency band ratios from the ISRUC database: (**a**) heart rate, (**b**) brain perfusion, (**c**) neural activity, and (**d**) synchronization.

A total of 14 features for each subject were evaluated. Comparing different training and testing subjects, mere accuracy changes were noted. Among SVM kernels, the polynomial 2 order kernel provided results with better accuracy (90%), sensitivity (100%), and specificity (83%) for the ISRUC database. The variation between sleep apnea and normal subjects is represented in the box plot, which shows the representation of the entropy feature for gamma, beta, alpha, theta, and delta bands from the ISRUC database (Figure 5a–e). Figure 6a–e shows the energy feature variation. Meanwhile, the heart rate, neural activity, brain perfusion, and synchronization feature variations are shown in Figure 7a–d. The proposed study documents that the finest features and increased database provided improved performance analysis compared to the existing literature [5,10,18].

**Figure 8:**
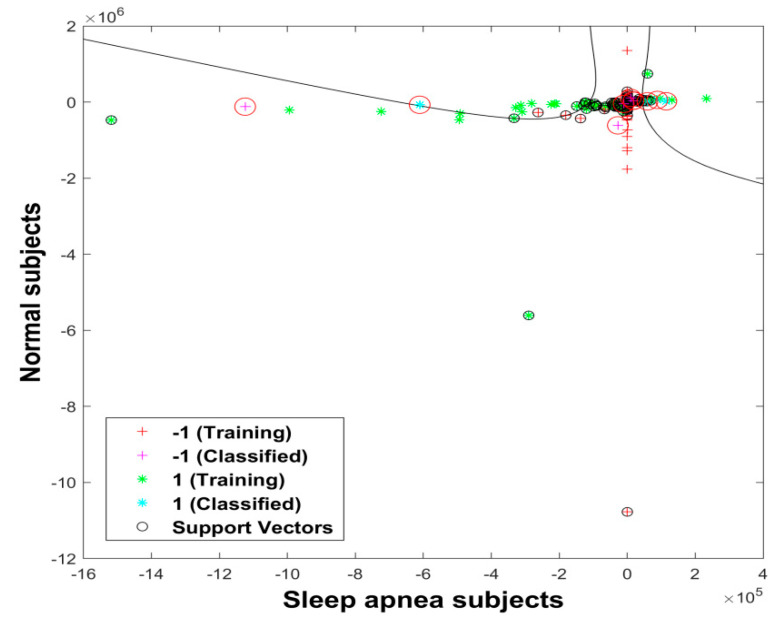
Classification of sleep apnea and normal subjects using the SVM classifier with polynomial order 2 kernel.

Furthermore, this study used a total of 150 trees for the RF classifier. Two thirds of the data were used for the training set and one third of the data were used for OOB validation. The accuracy, sensitivity, and specificity for the ISRUC, Sleep-EDF, and CAP Sleep databases obtained using this classifier were 66%, 100%, and 100%; 75%, 100%, and 100%; and 50%, 100%, and 100%, respectively. Most importantly, this study attempted the same experiments with the subjects of three databases and obtained improved results for the Sleep-EDF database. The RF classifier produced low-performance results when compared to the SVM classifier. The classification of normal and sleep apnea subjects using the SVM with a polynomial order 2 kernel from the ISRUC database is shown in Figure 8.

## 4. Discussion

A self-administered questionnaire was developed to analyze age, gender, smoking habits, and working hours to diagnose sleep apnea. In conclusion, the young, male adults, smokers, and those long working hours seemed to be highly affected by sleep apnea [34]. The diagnosis of sleep stages and sleep-related disorders were analyzed using the standard questionnaires from the analysis of age and gender. The power spectral density of different EEG frequency bands was measured in healthy individuals to assess sleep stages [15]. Recent literature has discussed the other EEG spectral parameters, i.e., the arousal index, central nervous system arousal, desynchronization, cognitive performance index, executive load index, performance enhancement index, LF-to-HF (low frequency to high frequency) ratio, task load index, and vigilance index to assess the dynamic workload condition for fighter pilots. [30].

In past decades, multiple studies have demonstrated the detection of sleep apnea using ECG signals with different classifiers. Contactless capacitive-coupled electrocardiography (ccECG) signals were used to detect sleep apnea by various signal quality indicators. Significant results were computed from HR and heart rate variability (HRV) features [2]. Another study used a single channel ECG-based OSA computer-aided design (CAD) using an optimal biorthogonal antisymmetric wavelet filter bank [5]. EEG signals are time-variable and noisy, which detects the electrical activity of the brain. In this proposed study, the classification of sleep apnea and normal subjects was performed using EEG signal analysis. Hence, extracting features from EEG signals was a challenging task that was accomplished by multiple conventional transforms. In comparison with conventional transforms, the WPD method is very competent at extracting the features from EEG signals. Ting et al. [35] emphasized the WPD method with an autoregressive model. Moreover, they concluded that results with better accuracy are obtained with the WPD method.

Rachim et al. [36] propounded a method that extracts features using the principal component analysis and SVM classifier. This method has been used in the fifth-level wavelet decomposition of features from ECG signals to detect sleep apnea. Ali et al. [37] performed level 4 WPD with an HRV signal to detect OSA. With the motivation of the existing literature, in this work, the WPD seemed to be used for band segregation and feature analysis and obtained an accuracy of 90%. This study validated the results with ratios of frequency band parameters that were explained by Nagendra et al. [21]. The yoga and control group was effectively reported with sub-band decomposition of EEG signals. Furthermore, frequency band ratios such as β/α, β/(α+θ), and 1/α were used to analyze the engagement task [38]. The results established improved performance results for utilizing such ratios in the proposed method.

In recent years, there has been little research focused on robust classifiers such as SVM and RF classifiers for an improved level of accuracy. Among the methods, SVM and ANN have been yielding promising results. Al-Angari et al. [39] demonstrated the SVM approach based on HRV, oxygen saturation, and respiratory signals to detect sleep apnea. The study results strongly recommend the SVM classifier with a kernel-based approach to improve performance results. Hence, better accuracy is achieved by the polynomial order 2 kernel. In addition, some of the literature provided better classification results using an RF classifier. Janbhakshi et al. [31] suggested using the HR and respiration rate from ECG signals to diagnose sleep apnea. The SVM and RF classifiers were used, which yielded 82% accuracy. With the motivation of the abovementioned literature, the proposed approach used the non-linear and ratio of frequency band parameters, which were significant and obtained enhanced results. In addition, this study utilized the SVM and RF classifiers for performance analysis and concluded that the SVM results in greater performance compared to the RF classifier. The novelty of the proposed approach is comparing the results among the three databases. The comparison result indicates that better improvement was achieved in subjects from the ISRUC database. An important limitation encountered in this approach is subject selection. The sleep apnea and normal subjects were directly chosen from the existing databases without any validation. Table 2 shows the summary of the existing literature related to this proposed study.

## 5. Conclusions

The normal and sleep apnea subjects were classified using SVM and RF classifiers with non-linear and sub-band decomposition of EEG signals. The classification was performed using SVM with three kernels: linear, radial basis function, and polynomial orders 2 and 3. From the analysis, it can be seen that SVM was the prominent method and outperformed with better accuracy using polynomial kernel (order 2) (90%). In conclusion, this study demonstrated that the chosen features seem to be effective between sleep apnea and normal subjects. Furthermore, this study can be used with real-time EEG signals for the detection and classification of sleep apnea.

## Figures and Tables

**Table 1:** Demographic characteristics of the ISRUC, Sleep-EDF, and CAP Sleep databases.

	ISRUC		Sleep-EDF		Cap Sleep	
	Subjects (M:F)	Age (Years)	Subjects (M:F)	Age (Years)	Subjects (M:F)	Age (Years)
Sleep subjects	57(33:24)	55 ± 14	20(15:5)	39 ± 18.54	4(4:0)	71.25 ± 7.22
Normal subjects	32(18:14)	45 ± 17	20(16:4)	33.15 ± 9.37	16(7:9)	32.18 ± 5.55
Training set	79	-	30	-	16	-
Testing set	10	-	10	-	4	-

Note: Data are represented as mean ± standard deviation, M:F—male:female.

**Table 2:** Summary of the performance comparison of existing literature.

Authors	Signals Used	Feature Used	Database	Subjects Used	Classifier Used	Event	Accuracy
Castro et al. [2]	ECG	Heart rate Heart rate variability	Volunteers	15	Signal quality indication	Sleep apnea	91.0%
Shrama et al. [5]	ECG	Fuzzy entropy, Log energy	Apnea ECG	27	Least square SVM	Sleep apnea	90.0%
Wang et al. [6]	ECG	RR intervals	Apnea ECG	35	Residual network	Sleep apnea	94.0%
Cui et al. [7]	EEG (F3-A2, C3-A2, O1-A2, F4-A1, C4-A1, and O2-A1)	Entropy	ISRUC	116	Convolutional neural network	Sleep stage classification (Wake, stage N1, stage N2, stage N3, and stage REM)	92.2%
Zhu et al. [8]	EEG (Pz-Oz)	Degree distribution, Horizontal visual graph, Difference visual graph	Sleep-EDF	8	Support vector machine	Sleep stage classification	87.5%
Tzimourta et al. [10]	EEG (F3-A2, C3-A2, O1-A2, F4-A1, C4-A1, and O2-A1)	Energy	ISRUC	100	Random forest	Sleep stage classification (Wake, stage N1, stage N2, stage N3, and stage REM)	75.3%
Savareh et al. [12]	EEG (Fpz-Cz and Pz-Oz)	Wavelet tree features	Sleep-EDF	61	Support vector machine, Artificial neural network	Sleep stage classification	90.3% ANN
Boostani et al. [13]	ECG	Entropy	Sleep-EDF	20	Random forest	Sleep apnea	87.1%
Elwali and Moussavi [16]	ECG	Optimized set of breathing sounds	PSG Sleep database at Misericordia Health Center (Winnipeg, Canada)	199	Random forest	Sleep apnea	81.4%
Aluhummadi et al. [18]	EEG	Energy, Variance	MIT-BIH	18	Support vector machine, Linear discriminant analysis, Naive Baiyes, Artificial neural network	Sleep apnea	97% SVM
Zhao et al. [19]	EEG (C3-A2 and C4-A1)	Sample entropy Variance	Tianjin Chest Hospital	30	Support vector machine, K nearest neighbor, Random forest	Sleep apnea	88.99% SVM
Saha et al. [20]	EEG (C3-A2 and C4-A1)	Inter band energy ratio δ-θ δ-α δ-σ δ-β θ-α	St. Vincent’s University Hospital/University College Dublin sleep apnea database	5	K nearest neighbor, Support vector machine, Linear discriminant analysis Naïve Bayes,	Sleep apnea	91.6% KNN
Tripathy et al. [32]	ECG	Heart rate Respiration signals	Apnea ECG	31	Support vector machine, Random forest	Sleep apnea	77.8% SVM
Rachim et al. [36]	ECG	Heart rate Respiration signals ECG-derived respiration	Apnea ECG	35	Support vector machine	Sleep apnea	95.0%
Ali et al. [37]	ECG	Heart rate variability	Sultan Qaboos University Hospital (SQUH)	80	Support vector machine	Obstructive sleep apnea	95.0%
Al-Angari et al. [39]	ECG	Respiration rate Oxygen saturation	Sleep Heart Health Study	100	Support vector machine	Obstructive sleep apnea	95.0%
Proposed study	EEG (C3-A2, Fpz-Cz, Pz-Oz, and C4-A1)	Entropy Energy Heart rate Synchronization Neural activity Brain perfusion	ISRUC, Sleep-EDF, CAP Sleep	159	Support vector machine, Random forest	Sleep apnea	90.0% SVM

## Data Availability

Data available in a publicly accessible repository. The data presented in this study are openly available in Physiobank ATM: https://archive.physionet.org/cgi-bin/atm/ATM (accessed on 4 June 2020).
